# Atherectomy in the Treatment of Peripheral Arterial Disease—A Case Series to Demonstrate Preferable Indications with Good Outcomes and a Literature Review

**DOI:** 10.3390/jcm14051437

**Published:** 2025-02-21

**Authors:** Marco Lizwan, Hao Yun Yap, Jack Kian Ch’ng, Tze Tec Chong, Nick Zhi Peng Ng

**Affiliations:** 1 Department of General Surgery, Singapore General Hospital, Singapore 169608, Singapore; 2 Department of Vascular Surgery, Singapore General Hospital, Singapore 169608, Singapore

**Keywords:** peripheral arterial disease, atherectomy, angioplasty, endovascular

## Abstract

**Background:** Endovascular therapy for lower-limb arterial disease is widely performed today. A vast array of sheaths, catheters, wires, balloon types, stents, and tools such as atherectomy, thrombectomy, and lithotripsy devices are now available to achieve the best outcomes in terms of vessel patency and ultimately limb salvage. The use of atherectomy devices, however, has raised some controversies in terms of outcome efficacy, cost effectiveness, and safety profile in various series and studies. Objectively, the types and disease pattern in these studies are also greatly heterogeneous. **Methods:** Here, we reported three cases which exemplify how these atherectomy devices have served as a valuable tool, especially for patients with complex and heavily calcified lesions. **Results:** The three cases highlighted scenarios where atherectomy displayed good outcomes, each involving the use of atherectomy devices to treat highly calcified vessels. **Conclusions:** Despite the concerns with atherectomy devices, we believe that with proper selection, patients will benefit most from their ability to achieve the best outcomes of both vessel patency and limb salvage.

## 1. Introduction

Peripheral arterial disease (PAD) poses a significant and growing challenge today due to the ageing population, rising prevalence of diabetes mellitus (DM), and end-stage renal disease (ESRD) in Singapore, mirroring global trends [1,2]. In this group of patients, PAD lesions are often heavily calcified, located in a small vessel, have poor outflow, and limited or no surgical bypass options [3]. These patients also have poor cardiac reserves [4] and are often not physiologically suited for surgical bypass revascularisation, or the nature of their disease makes open bypass unfeasible. As a result, endovascular therapy has emerged as a mainstay treatment, without ruling out bypass surgery as a form of bailout subsequently if needed.

Today, a wide array of endovascular therapies can be performed on a clinically driven target lesion. These include POBA (plain old balloon angioplasty), non-compliant POBA, POBA with DEB (drug-eluting balloon), POBA with stenting (bare, drug-coated, or bio-resorbable), atherectomy, and shockwave lithotripsy. As technology evolves, it is important to remember that the goals of therapy in PAD include pain relief, maintenance of ambulatory status, wound healing, limb preservation, and improved health-related quality of life [3], especially in patients with Rutherford 5 or 6 disease.

While almost every adjunctive therapy has studies showing better patency results compared to the original POBA, the larger question remains: how much better? Are these therapies cost-effective or justified? Recently, atherectomy devices have raised controversies regarding outcomes, efficacy, cost effectiveness, and safety in various studies. Further analysis reveals that the types and disease patterns in these studies are highly heterogeneous. This study aims to describe three cases from a vascular unit at a Singapore tertiary centre where atherectomy was beneficial. The outcomes of these interventions are influenced by operator experience, hospital, and resource availability.

## 2. Case Series

### 2.1. Case 1—Rotational Atherectomy for Severely Calcified Below-the-Knee (BTK) Steno-Occlusion with Chronic Limb-Threatening Ischemia (CLTI)

**Figure 1:**
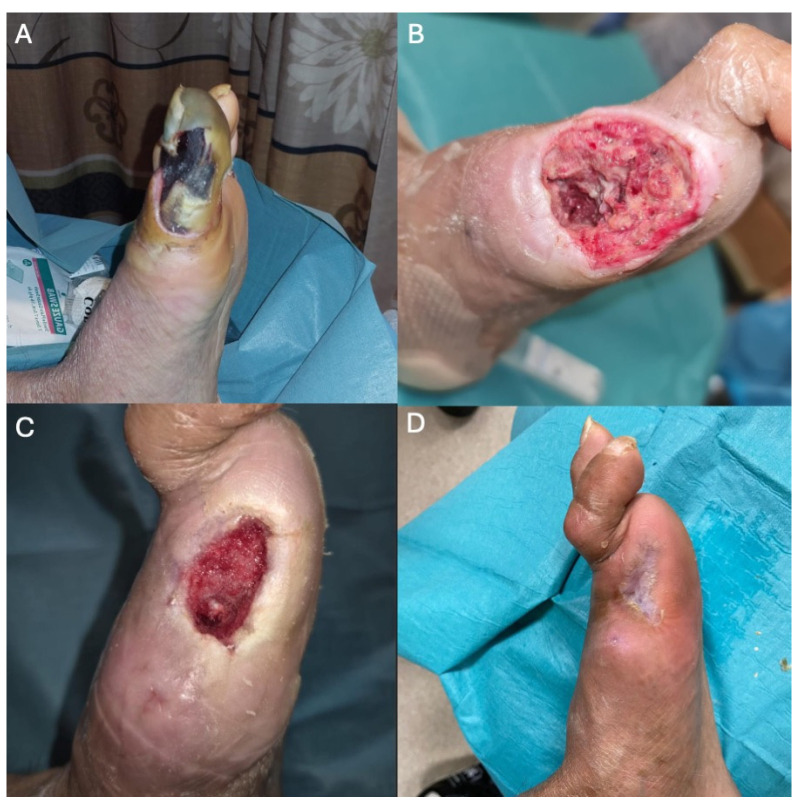
(**A**) Left first-toe wet gangrene at initial presentation. (**B**) First-toe ray amputation with good bleeding from raw edges of wound. Healing first-toe ray amputation wound at (**C**) 8 weeks and (**D**) 3 months post-angioplasty.

**Figure 2:**
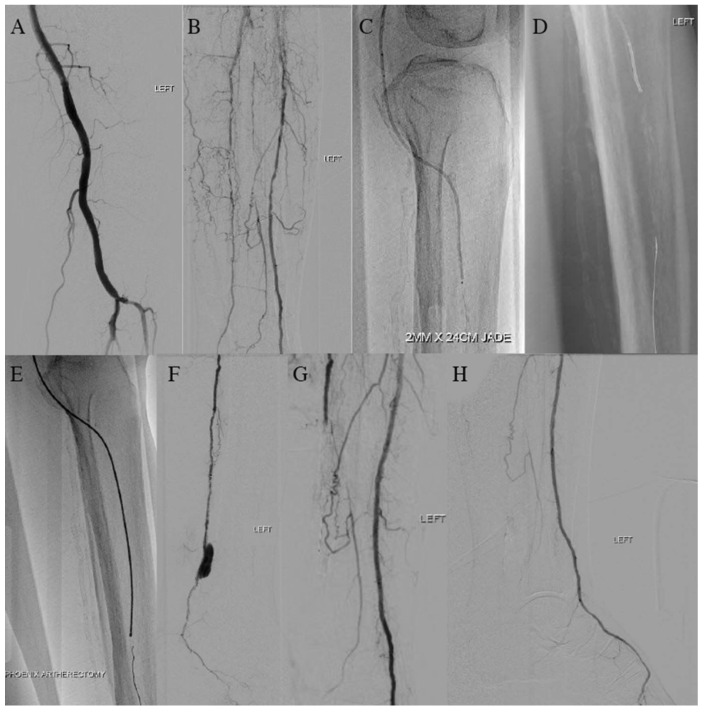
Intraoperative diagnostic angiography demonstrating (**A**) patent above-the-knee and tibioperoneal (TP) trunk arteries and (**B**) occlusion of anterior tibial artery (ATA) and posterior tibial artery (PTA). (**C**) Failed attempt to trace lesion with JADE 1.5. (**D**) Attempt at balloon deployment using forcible manner (BADFORM) for lesion; however, we were unable to track balloon. (**E**) Phoenix atherectomy of ATA occlusion. (**F**) Attempt at phoenix atherectomy of PTA occlusion but perforation was seen. (**G**,**H**) Post-procedure angiogram revealed good arterial flow to ATA and DPA.

A 62-year-old male with a previous medical history of hypertension, DM, and ESRD on peritoneal dialysis was referred for wet gangrene of the left first toe with absent pedal pulses (Figure 1A). Angiography revealed patent above-the-knee (ATK) femoropopliteal vessels with multilevel anterior tibial artery (ATA) and posterior tibial artery (PTA) steno-occlusion (Figure 2A,B). Successful wire crossing was achieved with the with Hi-Torque Command ES Guide Wire .014 (Abbott, Chicago, IL, USA). However, the severely stenosed and calcified proximal–mid segment of the ATA blocked the passage of the .018 CXI catheter (Cook Medical, Bloomington, MN, USA), JADE 1.5 (OrbusNeich, Hong Kong), and Armada XT 1.2 balloon (Abbott, Chicago, IL, USA)), despite multiple attempts at gradual proximal dilatation (Figure 2C).

The next consideration was to attempt retrograde wire crossing via the distal ATA and perform the BADFORM (balloon deployment using forcible manner) [5] technique (Figure 2D), but the retrograde wire could not transverse the lesion without going subintimal. At this point, only the antegrade Command ES .014 wire had seemingly crossed within the true lumen. Fortunately, it was able to be exchanged out for a .014 ViperCath XC Peripheral Exchange wire (Abbott, Chicago, IL, USA), allowing for debulking of the calcified lesion with the 6 Fr 2.2 Phoenix rotational atherectomy device (Philips, Baldwin Park, CA, USA) (Figure 2E).

A flow channel was successfully established, followed by angioplasty with a Jade 3 × 240 non-compliant .014 platform balloon (OrbusNeich, Hong Kong) and a SELUTION SLR sirolimus DEB (MedAlliance, Nyon, Switzerland). Satisfied with the ATA result, an attempt at wire crossing the PTA followed by atherectomy was performed, but a contained perforation was noted near the ankle (Figure 2F), and poor common plantar outflow led to the abortion of further revascularization. A palpable 2+ dorsalis pedis artery (DPA) pulse was felt at the procedure’s conclusion, with repeat angiograms showing good DPA outflow (Figure 2G,H).

The patient underwent first-toe ray amputation with good bleeding from the wound edges (Figure 1B). At routine follow-up, his wound was healing well, and he had a palpable 2+ DPA (Figure 1C,D).

### 2.2. Case 2—A Case of Rest Pain with In-Stent Chronic Occlusion

**Figure 3:**
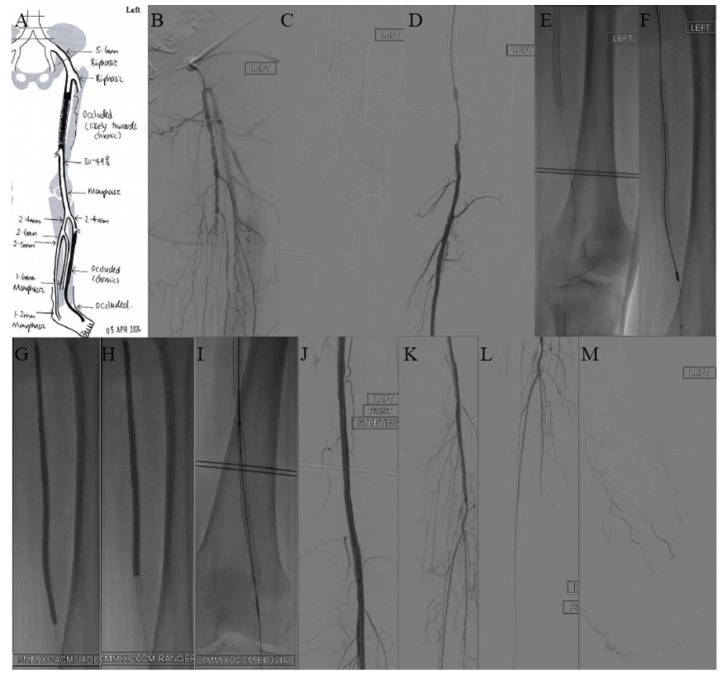
(**A**) Duplex ultrasound scan (DUS) showed long-segment chronic superficial femoral artery (SFA) in-stent occlusion. (**B**–**D**) Intraoperative diagnostic angiography showing chronic total occlusion from superficial femoral artery (SFA) to end of previous stent with some narrowing, of approximately 50%, noted at P1 area with possible dissection. (**E**) Embolic protection device was deployed at P1 to prevent distal embolization. (**F**) JETSTREAM atherectomy was performed. (**G**,**H**) Angioplasty performed. (**I**) Coverage of dissection flap by extending stent distally. (**J**–**M**) Post-procedure angiogram showed good result with no significant dissection, residual stenosis, or recoil.

A 65-year-old female smoker with DM, hypertension, dyslipidaemia, and ESRD on haemodialysis underwent SFA (superficial femoral artery) stenting three years ago for fifth-toe gangrene. She was subsequently lost to follow-up and re-presented to the clinic with worsening rest pain and reduced claudication distance. A duplex ultrasound scan (DUS) revealed long-segment chronic in-stent occlusion of the SFA (Figure 3A), with a right-toe pressure of 54 mmHg and left-toe pressure of 35 mmHg.

Angiography confirmed a 15 cm long in-stent chronic total occlusion extending from the SFA to the end of the previous stent, with reconstitution at the level of the abductor canal or P1 area (Figure 3B–D). The lesion was successfully crossed intraluminally with a V18 wire (Boston Scientific, Boston, MA, USA). An Emboshield NAV6 filter (Abbott, Chicago, IL, USA) was deployed at the P1 segment (Figure 3E), followed by Jetstream atherectomy (Boston Scientific, Boston, MA, USA) [6] (Figure 3F). Angioplasty was then performed with a JADE 5 × 240 mm (OrbusNeich, Hong Kong) and a DEB Ranger 6 × 150 mm (Boston Scientific, Illinois, USA) (Figure 3G,H). Upon retrieval of the filter, debris was noted. A decision was made to extend the stent distally to cover a dissection flap, using a 6 × 120 mm Eluvia stent (Boston Scientific, Boston, MA, USA) (Figure 3I), followed by balloon moulding with Mustang 6 × 120 mm (Boston Scientific, Boston, MA, USA).

A repeat angiogram demonstrated good results with no significant dissection, residual stenosis, or recoil (Figure 3J–M) and palpable 2+ pedal pulses were present. The patient reported an improvement in her claudication and rest pain. At her 3-month follow-up, she continued to have palpable 2+ DPA and PTA.

### 2.3. Case 3—A Case of Severely Calcified Femoral–Popliteal Disease with Extensive Circumferential Calf Wounds

**Figure 4:**
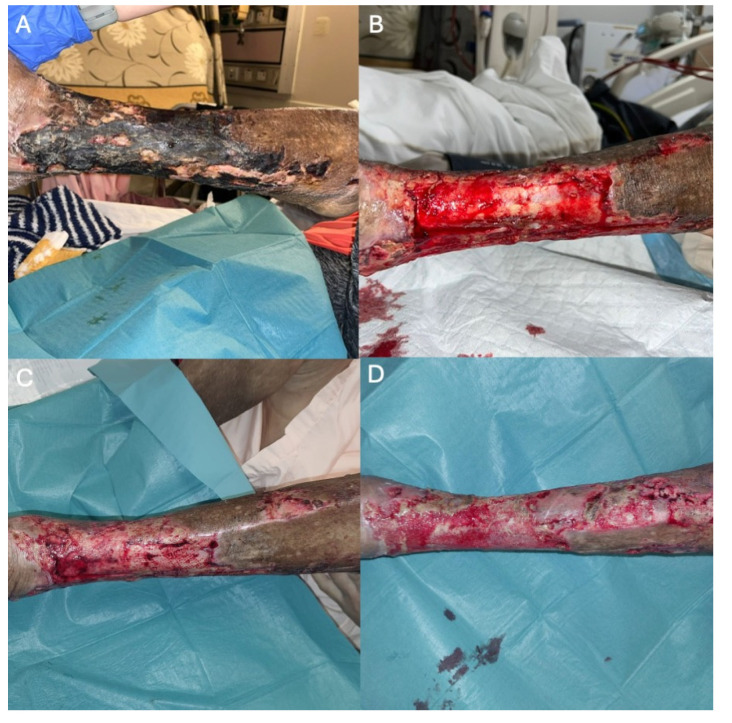
(**A**) Superficial wound with developing eschar seen on her left lateral lower shin during initial presentation. (**B**) Left lower-limb wound debridement with good bleeding after. (**C**,**D**) Healing of wound during inpatient stay.

**Figure 5:**
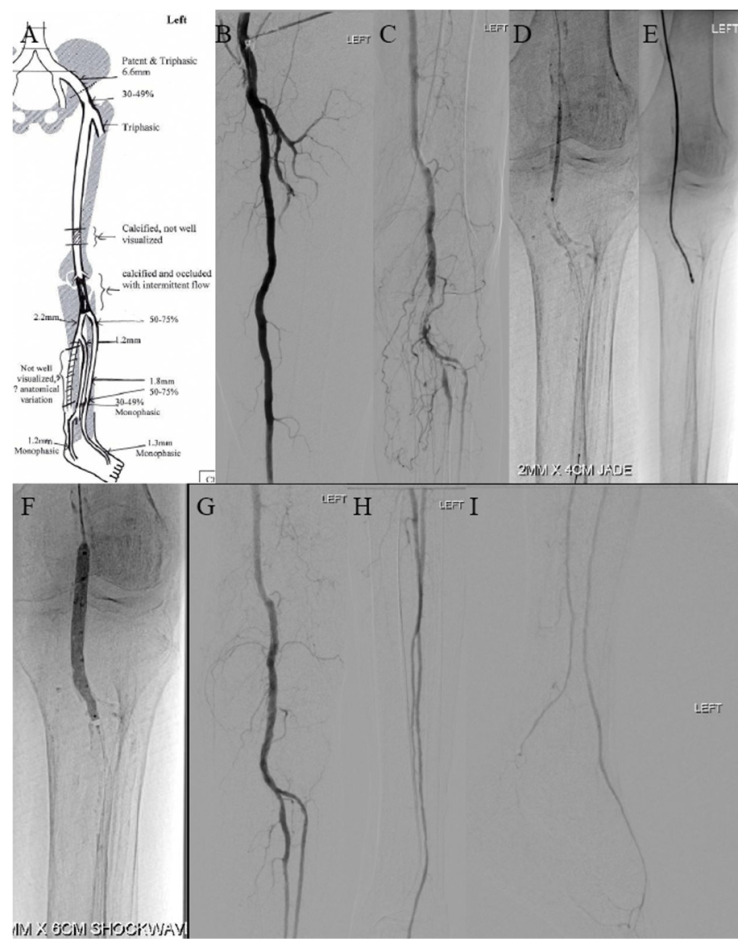
(**A**) DUS showed moderate to severe calcification throughout arterial system of left lower limb. (**B**,**C**) Intraoperative diagnostic angiography demonstrated patent common femoral artery with occlusion of femoro-popliteal vessels from P1 to P3 area. (**D**) Difficulty to track JADE 2 due to tight occlusion. (**E**) Phoenix atherectomy was performed. (**F**) Shockwave intravascular lithotripsy was performed with 4.5 × 60 mm Shockwave M^5+^ balloon. (**G**–**I**) Post-procedure angiogram showed good result with no significant dissection, residual stenosis, or recoil.

A 62-year-old female smoker with DM, hypertension, and ESRD on haemodialysis and SLE (systemic lupus erythematosus) on long-term hydroxychloroquine and prednisone presented with left lower-limb PAD. A superficial wound with developing eschar was seen on her left lateral shin (Figure 4A). DUS revealed moderate to severe calcification throughout the arterial system of her left lower limb (Figure 5A), with right-toe pressure >230 mmHg and a left-toe pressure of 54 mmHg.

Angiography showed a patent common femoral artery but occlusion of the femoropopliteal vessels from the P1 to P3 area (Figure 5B,C), along with multilevel stenosis and occlusion in the ATA and peroneal artery. The lesion was crossed intraluminally with the Victory 25 tip wire (Boston Scientific, Boston, MA, USA), supported by a .018 CXI catheter (Cook Medical, Bloomington, MN, USA). Tracking the JADE 2 (OrbusNeich, Hong Kong) (Figure 5D) and 4.5 Shockwave M^5+^ (Shockwave Medical, Santa Clara, CA, USA) was challenging due to focal calcification. Given the tight occlusion, atherectomy was performed using a 2.2 Phoenix rotational atherectomy device (Philips, Baldwin Park, CA, USA), after exchanging the wire for a 0.014 ViperCath XC Peripheral Exchange wire (Abbott, Chicago, IL, USA) (Figure 5E). A good flow channel was achieved after the atherectomy, but due to the heavy calcification, shockwave intravascular lithotripsy was performed using a 4.5 × 60 mm Shockwave M^5+^ balloon, delivering 300 pulses (Figure 5F). Subsequent angioplasty was performed with the DEB Ranger 5 × 240 mm (Boston Scientific, MA, USA). No stenting was required for the femoropopliteal vessels, as good effacement was achieved without dissection following shockwave lithotripsy and DEB angioplasty.

Angioplasty was also performed for the ATA and peroneal artery steno-occlusion. Repeat angiogram showed a good result with no significant dissection, residual stenosis, or recoil (Figure 5G–I) and palpable 2+ pedal pulses. She underwent left lower-limb wound debridement with good bleeding after (Figure 4B). Her wound underwent inpatient monitoring and was healing well during her stay (Figure 4C,D).

## 3. Discussion

Three cases where atherectomy played a crucial role were presented, each involving the use of atherectomy devices to treat highly calcified vessels in patients with ESRD. In Singapore, the prevalence of DM, ESRD, and PAD is on the rise [7]. DM and ESRD are well-established independent risk factors for the development of CLTI [8], and patients with these conditions frequently present with heavily calcified PAD lesions [3]. A recent study has shown that there are differences in diabetes progression and complication risk in Asian populations as compared to those of European ancestry. In Singapore, many DM patients are at high risk of progressing to symptomatic PAD, ulceration, infection, and even gangrene, potentially requiring amputation [9].

Endovascular therapy for lower-limb arterial disease is well established. Although atherectomy devices vary in their design, their primary goal is to effectively debulk calcified plaque, improving vessel compliance and facilitating subsequent interventions such as POBA, DEB angioplasty or vessel preparation for stenting. In some cases, atherectomy may help to avoid the need for stenting altogether by reducing the risk of dissection after balloon angioplasty. In Singapore, atherectomy is often used for treatment of advanced Rutherford 4–6 lesions, which present with tissue loss and gangrene, due to the higher burden of severe disease in our local population [10].

The first case demonstrates the use of atherectomy to enhance vessel crossability. In a severely calcified BTK lesion, where even the crossing support catheter and a small-size balloon were unable to traverse the lesion, the atherectomy device proved invaluable. Such aggressive therapy is warranted particularly in this instance with wet gangrene as vessel patency to achieve limb salvage is paramount for the patient to avoid a major limb amputation.

The case also demonstrates two downsides. Firstly, it was fortunate that the softer antegrade ES Command Wire could be exchanged for a Viper Wire even though the crossing support catheter could not transverse the lesion for exchange. Performing atherectomy over a soft Command ES .014 wire risks fragmenting the wire within the true lumen. Secondly, overzealous or careful sizing of the atherectomy device is important. Otherwise, there is a risk of perforation as seen with the PTA.

It is also crucial to perform atherectomy in the “true” lumen rather than subintimally. Although shockwave lithotripsy can be performed in a non-true lumen vessel, BTK experience with shockwave is limited, particularly when pre-dilation with a smaller balloon is not possible. Many of these vessels are also at higher risk of complications in CLTI due to the severity of the disease and the poor condition of the blood vessels.

The second case demonstrates a chronic SFA in-stent occlusion with debilitating rest pain in a patient who is unable to quit smoking. Stents can be seen as a necessary evil especially for patients who continue to smoke. When acutely occluded or when the occlusion is subacute, this occlusion can be easily recanalized; however, chronic occlusion proves a real challenge to achieve meaningful luminal gain [11]. The case also demonstrated the use of the Jetstream atherectomy with a distal filter to prevent distal emboli. The lumen is subsequently treated with drug elution balloon as recommended by the JET-RANGER study [12].

Furthermore, the second case highlights the role of duplex stent surveillance. While not evidence-based, is perhaps crucial so that these lesions can be treated before they are completely occluded [13]. Follow-up care is also crucial to ensure these patients are compliant to smoking cessation and antiplatelet or anticoagulation therapy. Certainly, a femoral–popliteal bypass would also be possible if the popliteal outflow has not been covered by a prior stent and the patient is surgically fit with the availability of a conduit.

In the third case, a patient with an extensive Rutherford 6 wound as well as severe popliteal calcified occlusion as a result of ESRD and long-term steroid use for treatment of SLE was presented. Long-term steroid usage is known to contribute to accelerated atherosclerosis, poor wound healing, and immunosuppression, as well as challenging glycaemic control. As the patient is only in her early 60s and premorbidly ambulant, she was adamant that she wanted limb salvage. As the lesion was at the popliteal artery across the knee joint, the decision was made to use a combination of atherectomy and shockwaves to achieve the best luminal gain without dissection in hope of avoiding stenting across the knee joint. While stents with a high radial force are available for stenting across the knee joint, given the repeated stress, these stents are at a high risk of needing early reintervention [14].

### 3.1. Atherectomy as Treatment Modality for PAD

Recent studies have placed atherectomy under significant debate and scrutiny, particularly regarding its impact on long-term outcomes such as patency and limb salvage. While the DEFINITIVE-LE Study reported that atherectomy followed by drug-coated balloon (DCB) angioplasty resulted in a primary patency rate of 90.5% at year one, which is significantly higher than POBA alone [15], other studies have shown no significant benefit over POBA or stenting in terms of long-term patency [16,17,18]. Complications such as vessel perforation (as in our first case), restenosis, and distal embolization have also been major concerns with the use of atherectomy [19].

Furthermore, atherectomy is more expensive compared to other endovascular treatments; hence, its cost-effectiveness has been heavily scrutinized. The additional cost raises multiple questions, especially when outcomes are similar to other less expensive endovascular therapies [17]. 

Atherectomy is an effective endovascular technique for removing atherosclerotic plaque in patients with PAD; however, its use is associated with several challenges. One of the most concerning complications is peripheral embolization, with reported incidence rates ranging from 3% to 30%, depending on the device type and lesion characteristics. Directional atherectomy has been associated with higher embolization rates compared to rotational, orbital, and laser atherectomy [20]. The mechanism of embolization is primarily due to plaque fragmentation and downstream migration of debris, which can lead to distal ischemia and the no-reflow phenomenon. To mitigate this risk, embolic protection devices (EPDs) have been utilized, particularly in high-risk lesions, alongside strategies such as pre-dilatation with balloon angioplasty, controlled and gradual device passes, and aspiration catheters [21]. Additionally, careful patient selection and operator expertise are crucial in optimizing outcomes and reducing complications. While atherectomy remains a valuable tool in peripheral revascularization, further studies are needed to refine techniques, improve embolic protection, and identify patient populations that would benefit the most from this approach.

### 3.2. Heterogeneity in Evidence for Atherectomy

Evidence for the use of atherectomy in PAD is highly heterogenous, reflecting diverse outcomes across various studies. This variability can be attributed to differences in study designs, patient populations, lesion characteristics, and the types of atherectomy devices used (Table 1). The heterogeneity in evidence underscores the need for individualized treatment plans and highlights the importance of ongoing research to better define the role of atherectomy in the management of PAD.

### 3.3. Atherectomy in Treating Different Stages of PAD

Atherectomy can be used to treated different stages and symptoms of PAD. The effectiveness of atherectomy can vary depending on the clinical presentation and severity of the disease.

Atherectomy is used in claudication, an early symptom of PAD, to improve exercise tolerance and alleviate symptoms by removing plaque and restoring blood flow. However, when compared to medical management and supervised exercise therapy, atherectomy for claudication has not demonstrated superior long-term outcomes [35]. On the other hand, revascularization for claudication has been associated with worse clinical outcome, including an increased rate of disease progression and amputation [36,37].

Rest pain is a more severe symptom of PAD. The goal of atherectomy in rest pain is to relieve pain by improving blood flow and reducing arterial blockages. The use of atherectomy for rest pain is less studied as compared to claudication and CLTI.

Atherectomy is commonly used in CLTI—the most severe form of PAD—characterized by non-healing wounds, ulcers, or gangrene and is associated with a high risk of limb loss. The need for aggressive revascularization makes atherectomy a good option of endovascular therapy as atherectomy is often used in cases with heavily calcified lesions where other treatments might fail.

### 3.4. ATK Versus BTK Atherectomy

There are differences in the use of atherectomy above and below the knee, primarily due to the anatomical and physiological variations in these regions. In ATK atherectomy (femoral and popliteal arteries), lesions are often more complex and may involve a mix of calcified and fibrous tissue [38]. Meanwhile, the arteries in the BTK region (such as tibial arteries) can be more challenging due to the smaller vessel size and greater plaque calcification [38]. Atherectomy can be helpful but there is a greater risk of vessel injury and procedural complications in these smaller, more delicate vessels.

In ATK lesions, atherectomy is often associated with favourable outcomes in terms of reducing restenosis rates and improving long-term patency when performed in larger, more accessible vessels [39]. Meanwhile, in BTK lesions, outcomes are more variable. While atherectomy can improve results, it must be carefully managed to avoid adverse effects [40]. In some cases, other treatments or a combination of therapies may be considered.

### 3.5. Recommendation for Atherectomy

There is no clear consensus on the ideal patient population or lesion characteristics for atherectomy. While it is often employed in cases with severe calcifications where other modalities have failed, comprehensive guidelines for its use are lacking [41].

Atherectomy is typically considered for patients with CLTI who have heavily calcified lesions, significant plaque burden, or failed previous endovascular therapies. When dealing with calcified or hard lesions, atherectomy can help by shaving off or cutting into the calcified lesions. This can help to better prepare the vessel for subsequent interventions for subsequent intervention, such as POBA or stenting, and potentially reduce the risk of dissection and the need for stenting. By reducing the plaque burden and improving vessel compliance, atherectomy can make these procedures more effective and safer.

Additionally, in the treatment of in-stent restenosis (ISR), atherectomy is also often preferred over BA for several reasons. Atherectomy can remove or reduce the thickness of the restenotic tissue, which is typically fibrous or calcified. By reducing the plaque burden and modifying the lesion, it can help improve the effectiveness of subsequent balloon angioplasty or stenting [42]. Atherectomy also helps to minimize complications such as dissection or recoil by making the vessel wall smoother. If stenting is needed after atherectomy, it can lead to better stent expansion and apposition compared to BA alone [43].

## 4. Conclusions

Atherectomy remains a valuable tool in the management of PAD, particularly in patients with complex and heavily calcified lesions. While it shows potential for improving primary patency and limb salvage rates, its use should be carefully considered, given the associated risks and higher costs. Combining atherectomy with other endovascular techniques can enhance patient outcomes. Further research and well-designed clinical trials are needed to better define its role and indications as treatment strategies for PAD.

Moreover, the profile of the local Singapore patient population justifies the use of atherectomy in PAD, as it has the potential to improve limb salvage rates and quality of life while reducing long-term healthcare costs associated with major amputations and extended wound care.

## Figures and Tables

**Table 1:** Literature reviews of the use of atherectomy to treat peripheral arterial disease.

Reference	Year	Technique	Study Population	Arteries	Conclusion
Safian et al. [22]	2009	Orbital atherectomy (OA)	Claudication or CLTI	Infrapopliteal	OA is safe and effective in the short-term
McKinsey et al. [15]	2014	Directional atherectomy (DA)	Claudication or chronic limb-threatening ischemia (CLTI), lesion length up to 20 cm	Infrainguinal	DA is safe and effective
Roberts et al. [23]	2014	DA	8–36 cm vessel stenoses or occlusions with bilateral vessel wall calcification	Femoropopliteal	DA is safe and effective to treat moderate to severely calcified femoropopliteal arteries when used with distal embolic protection device
Garcia et al. [24]	2015	DA	Claudication, Rutherford 1–3	Infrainguinal	DA is effective in diabetic and non-diabetic claudicants
Rastan et al. [25]	2015	DA	Claudication or CLTI, lesion length up to 20 cm	Infrapopliteal	DA is safe and effective
Giannopoulos et al. [26]	2020	OA	Claudication or CLTI	Infrainguinal	Balloon angioplasty with adjunctive OA is safe and effective
Giannopoulos et al. [27]	2021	Various endovascular therapies	CLTI, Rutherford 2–6	Infrainguinal	Atherectomy combined with other therapies had better outcomes when compared to atherectomy alone
Giusca et al. [28]	2021	Phoenix atherectomy (PA)	Claudication, rest pain, or CLTI	Infrainguinal	PA in combination with DCB is safe and effective in complex, calcified peripheral lesions
Rocha-Singh et al. [29]	2021	DA	Moderate to severely calcified lesions	Femoropopliteal	DA prior to DCB angioplasty is safe and effective in patients with symptomatic severely calcified femoropopliteal PAD prior to DCB angioplasty
Shammas et al. [30]	2021	Jetstream atherectomy (JA)	In-stent restenosis (ISR)	Femoropopliteal	JA without drug-eluting device is feasible to treat femoropopliteal ISR
Giusca et al. [28]	2022	PA	Claudication or CLTI	Infrainguinal	PA is safe in patients with complex lesions
Shammas et al. [12,31]	2022	JA	Claudication or rest pain/ulcerations	Superficial femoral artery/popliteal artery	JA demonstrated higher initial technical success, but long-term patency rates were comparable to balloon angioplasty
	2023				
Pan et al. [32]	2022	Rotarex atherectomy (RA)	Total in-stent occlusion	Femoropopliteal	RA with drug-coated balloon (DCB) is safe and effective in patients with femoropopliteal total in-stent occlusion
Dukic et al. [33]	2023	JA	CLTI, Rutherford 1-5	Femoropopliteal	JA with DCB is safe and effective in complex and calcified femoropopliteal lesions
Schöfthaler et al. [34]	2024	PA	Claudication or CLTI	Common femoral artery/popliteal artery	PA is safe and effective. When PA is combined with DCB, there is higher success compared to DCB alone

## Data Availability

Additional data are available from the corresponding author on reasonable request.

## Abbreviations

The following abbreviations are used in this manuscript:

PAD	Peripheral arterial disease
DM	Diabetes mellitus
ESRD	End-stage renal disease
POBA	Plain old balloon angioplasty
DEB	Drug-eluting balloon
BTK	Below the knee
CLTI	Chronic limb-threatening ischemia
ATK	Above the knee
ATA	Anterior tibial artery
PTA	Posterior tibial artery
BADFORM	Balloon deployment using forcible manner
DPA	Dorsalis pedis artery
SFA	Superficial femoral artery
DUS	Duplex ultrasound scan
SLE	Systemic lupus erythematosus
DCB	Drug-coated balloon
OA	Orbital atherectomy
DA	Directional atherectomy
PA	Phoenix atherectomy
JA	Jetstream atherectomy
RA	Rotarex atherectomy
